# UPLC-ESI-MS/MS Profiling of Secondary Metabolites from Methanol Extracts of In Vivo and In Vitro Tissues of *Daucus capillifolius* Gilli (A Comparative Study)

**DOI:** 10.3390/molecules29112694

**Published:** 2024-06-06

**Authors:** Rehab H. Abdallah, Wafaa H. B. Hassan, Shaza M. Al-Massarani, Wael M. Abdel-Mageed, Samih I. Eldahmy, Omer A. Basudan, Mehtab Parveen, Entesar El Senosy, Sahar Abdelaziz

**Affiliations:** 1 Department of Pharmacognosy, Faculty of Pharmacy, Zagazig University, Zagazig 44519, Egypt; rehabhamed2000@yahoo.com (R.H.A.); wafaahbh@zu.edu.eg (W.H.B.H.); elsameeh@gmail.com (S.I.E.); drintessar2015mohsen@yahoo.com (E.E.S.); 2 Department of Pharmacognosy, College of Pharmacy, King Saud University, P.O. Box 2457, Riyadh 11451, Saudi Arabia; salmassarani@ksu.edu.sa (S.M.A.-M.); basudan@ksu.edu.sa (O.A.B.); 3 Department of Chemistry, Faculty of Science, Aligarh Muslim University, Aligarh 202002, UP, India; mehtab.organic2009@gmail.com

**Keywords:** *Daucus capillifolius*, UPLC-ESI-MS/MS, callus, phenolic acids, flavonoids, acetylenic compounds

## Abstract

*Daucus capillifolius* Gilli is a rare annual wild herb grown in Libya. It belongs to the Apiaceae family, which is one of the largest flowering plant families. Plants of this family are outstanding sources of various secondary metabolites with various biological activities. A UPLC-ESI-MS/MS analysis of different extracts of in vivo and in vitro tissues of *Daucus capillifolius* together with the fruit extract of the cultivated plant in both ionization modes was carried out for the first time in the current study. Our results reveal the tentative identification of eighty-seven compounds in the tested extracts, including thirty-two phenolic acids and their derivatives; thirty-seven flavonoid glycosides and aglycones of apigenin, luteolin, diosmetin, myricetin and quercetin, containing glucose, rhamnose, pentose and/or glucuronic acid molecules; seven anthocyanins; six tannins; three acetylenic compounds; and three nitrogenous compounds. The tentative identification of the above compounds was based on the comparison of their retention times and ESI-MS/MS fragmentation patterns with those previously **reported** in the literature. For this Apiaceae plant, our results confirm the presence of a wide array of secondary metabolites with reported biological activities. This study is among the first ones to shed light on the phytoconstituents of this rare plant.

## 1. Introduction

Recently, great attention has been paid to the chemical and biological investigation of native medicinal plants, which constitute a gold mine of phytoconstituents with exceptional biological activities and represent an essential source of novel bioactive drugs. The extraction and isolation of targeted, safe and potent antimicrobial natural drugs is becoming of vital importance to control the microbial resistance to reported chemically synthesized drugs and food deterioration resulting from fungal or bacterial infections. North Africa is still a very rich source of untapped medicinal plants that are undergoing extensive screening for novel natural drug discovery [1].

The Apiaceae family (previously Umbelliferae), commonly referred to as carrot or parsley family, comprises approximately 3780 species and 434 genera distributed in temperate zones. It includes various herbs and vegetables of variable medicinal and economical importance [2,3]. Notably, plants of this family are a rich source of specialized secondary metabolites (furanocoumarins, sesquiterpene lactones and sesquiterpene coumarins) [4] with various biological activities, such as antimicrobial, anti-cancer, and cyclooxygenase inhibitory activities [5]. Moreover, these plants are distinguished for their diverse uses, serving purposes in food, flavorings (spices and condiments), decorative applications and medicinal practices and contributing significantly to the food, fragrance, pharmaceutical, cosmetic and cosmeceutical industries [3,6,7,8,9,10,11].

Numerous societies, including the ancient Egyptian, Mexican, Indian and Chinese, were accustomed to Apiaceae plants. Plants of this family also contain a mixture of aliphatic C_17_ polyacetylenes, including falcarinol, the most bioactive polyacetylene present in this family. Falcarinol has cytotoxic activity against human gastric adenocarcinoma, as well as other possible beneficial effects, such as anti-inflammatory and anticoagulant properties. In addition, polyacetylenes are well-known antifungal compounds [2,12].

In addition, flavonoids and anthocyanins are commonly found in the Apiaceae family and exhibit a diverse range of biological activities. Flavonoids are renowned for their protective role in treating conditions such as carcinogenesis, inflammation, and atherosclerosis. They also exhibit diverse properties, including antiviral, antimicrobial, antihepatotoxic, antiosteoporotic, antiulcer, immunomodulatory, antiproliferative and high antioxidant capacity [13]. Recently, it was reported that *Petroselinum crispum* from Apiaceae showed significant antioxidant and estrogenic activities due to the presence of phytoestrogens like diosmetin and apigenin [14]. Similarly, anthocyanins demonstrate a diverse array of biological activities encompassing antioxidant, anti-inflammatory, antimicrobial and anti-carcinogenic effects.

Additionally, they play a role in enhancing vision, inducing apoptosis, providing neuroprotection, and influencing blood vessels and platelets, which may potentially reduce the risk of coronary heart disease [15]. Genus *Daucus* (Apiaceae) contains about 60 species distributed mainly in Africa, West Asia, and Europe, and a few species were found in Australia and North America [16,17]. It is represented in Libya by nine species, including *D. durieua*, *D. muricatus*, *D. carota*, *D. guttatus*, *D. capillifolius*, *D. jordanicus*, *D. littorals*, *D. syrticus* and *D. sahariensis* [18]

Chemically, *Daucus* is one of the richest sources of volatile oil, sterols and triterpenes, polyacetylenic compounds, flavonoids and sesquiterpene lactones. Biologically, the volatile oil of many species of this genus showed a wide range of important pharmacological activities, such as antioxidant, cytotoxicity, insecticidal, antimicrobial and anti-inflammatory activities [19]. *Daucus capillifolius* Gilli from Libya is a rare annual wild herb with an erect and smooth stem which reaches about 50 cm in length [18]

Based on the available literature, nothing has been reported on the phytochemical constituents of this plant, except our previous work, which investigated the micropropagation and callus culture of this endangered plant in addition to the GC-MS analysis of its essential-oil constituents [19]. The current study aimed to conduct a comparative phytochemical investigation of the methanolic extracts of in vivo (cultivated fruit) and in vitro tissues (calli grown on different media with various hormonal combinations) of this rare plant by using UPLC-ESI-MS/MS analysis in both ionization modes.

## 2. Results and Discussion

**Figure 1:**
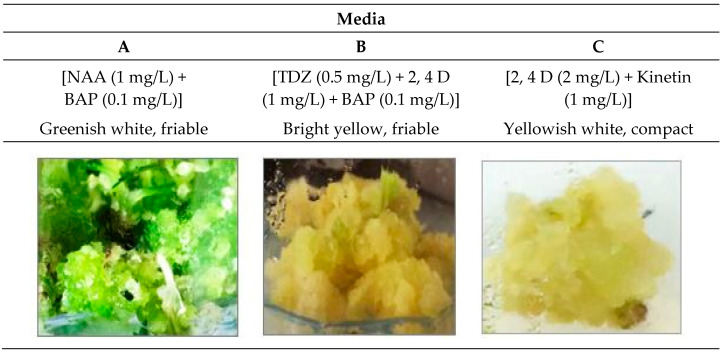
Morphological characters of callus produced from leaf explants of *D. capillifolius* Gilli on different culture media (media A, B &C) after 40 days of cultivation.

**Figure 2:**
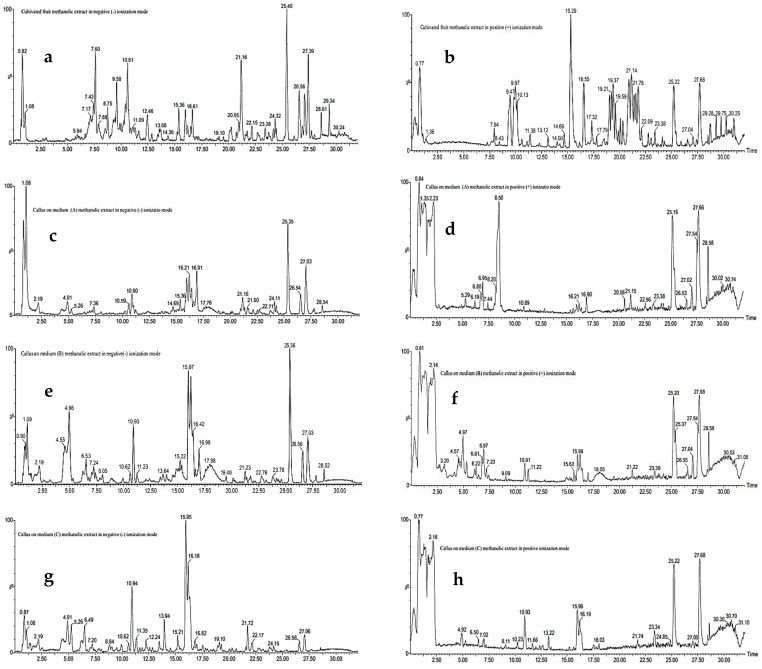
LC-MS total ion chromatograms (TICs) of methanolic extracts of *Daucus capillifolius* Gilli cultivated fruits in negative ionization mode (**a**,**c**,**e**,**g**) and positive ionization mode (**b**,**d**,**f**,**h**).

The methanolic extracts of in vivo (the fruit extract of the cultivated plant) and in vitro tissues of *D. Capillifolius* Gilli (Figure 1) were analyzed by using UPLC-ESI-MS/MS. The analysis revealed the tentative identification of 87 different phenolic and non-phenolic compounds (Figure 2).

### 2.1. Identification of Phenolic Compounds of Methanol Extracts of In Vivo and In Vitro Tissues of D. capillifoliusby UPLC–ESI-MS/MS

Eighty-one compounds were tentatively identified for the first time in the methanol extracts of in vivo and in vitro tissues of *Daucus capillifolius* fruits. The results are illustrated in Table 1, Table 2 and Table 3.

#### 2.1.1. Identification of Phenolic Acids and Acid Derivatives in Methanol Extracts of In Vivo and In Vitro Tissues of *D. capillifolius* Gilli

As shown in Table 1, thirty-two phenolic acids and their derivatives were identified in methanol extracts of in vivo and in vitro tissues of *D. capillifolius* Gilli as follows.

Compound **1** was suggested to be malic acid due to the presence of the molecular ion peak at *m/z* of 133 [M + H]^+^ [20].

Compounds **2** and **25** showed the same pseudo-molecular ion peak at *m/z* of 279 [M-H]^−^ and were suggested to be benzoic acid and coumaric acid derivatives, respectively. This suggestion was based on the presence of MS^2^ base peak fragment ions at *m/z* of 121.0 and 162.6, respectively [21].

Compound **3** exhibited a deprotonated molecular ion peak at *m/z* of 329. Based on the presence of one main fragment in the MS^2^ spectrum at *m/z* of 131.1 [pentose-H], which resulted from a neutral loss of the syringic acid molecule [M-H−198]^−^, it was identified as syringic acid pentoside, which has not been reported in *Daucus* species [22].

Compound **4** had a precursor ion at *m/z* of 327 [2M-H]^−^ and a protonated molecular ion at *m/z* of 165. In negative ESI mode, the MS^2^ showed a base peak fragment ion at *m/z* of 163.7 for [M-H]^−^, so it was identified as coumaric acid [23]. Coumaric acid was previously isolated from the genus *Daucus* [24].

Compounds **5** and **7** had deprotonated molecular ions at *m/z* of 137 and an MS^2^ base fragment ion at *m/z* of 92.9. Based on the mass fragmentation and the low retention time, as well as previously published reports, these compounds were tentatively identified as hydroxybenzoic acid, which was previously detected in *Daucus* [25,26].

Compounds **6** and **9** were identified as gallic acid derivatives according to the LC-MS^1^ and MS^2^ data reported in Table 1**.** Similarly, compounds **28** and **31** were tentatively identified as benzoic acid and quinic acid derivatives, respectively [21].

**Figure 3:**
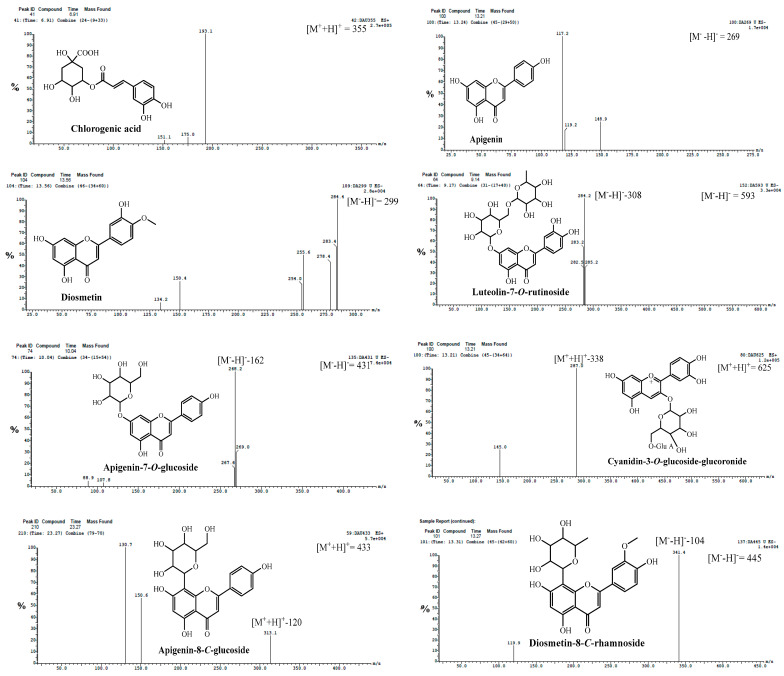
Fragmentation pattern mass spectra of some identified phenolic compounds from methanolic extracts of cultivated fruits and calli grown on different media of *Daucus capillifolius* Gilliin in positive (+) and negative (−) ionization modes.

Compound **8** had a molecular ion fragment at *m/z* of 179 [M-H]^−^ and an MS^2^ base fragment ion at *m/z* of 135.2 [M-H-COOH] (Figure 3). Based on mass fragmentation, as well as previously reported data [25], compound **8** was identified as caffeic acid. It was previously isolated from the genus *Daucus* [24].

Compound **10** showed a deprotonated molecular ion peak at *m/z* of 279 [M-H]^−^. It was suggested to be a vanillic acid derivative based on the presence of the MS^2^ base peak fragment ion at *m/z* of 167.4 **(**Table 1) [22].

Compounds **11, 17** and **29** had deprotonated and protonated molecular ions at *m/z* of 353 and 355, respectively (Figure 3). They had MS^2^ fragment ions at *m/z* of 191.3 with a relative abundance of 100%. This fragmentation pattern was found to be consistent with previous findings on chlorogenic acid reported by [25]. Notably, chlorogenic acid has been detected in *Daucus* species, as mentioned by [26].

Compound **12** with a molecular ion compound at *m/z* of 329 [M^−^-H]^−^ was tentatively identified as a cinnamic acid derivative. The HPLC-ESI-MS spectra of this compound showed an MS^2^ base peak fragment ion at *m/z* of 146.6 for the cinnamic acid moiety after the loss of 182 *amu* [22].

**Table 1:** Phenolic compounds tentatively identified in in vivo and in vitro tissues of *D. capillifolius* Gilli by using UPLC-ESI-MS/MS analysis in positive (+) and negative (−) ionization modes.

No.	R_t_ (min)	Name	Parent Ion (*m/z)*	MS^2^ Fragments *(m/z)*	I	II	III	IV	Reference
1	0.87	Malic acid	133		−	−	4.92	−	[20]
2	1.92	Benzoic acid derivative	279	121.0 (100%)	−	+	−	−	[21]
3	1.96	Syringic acid pentoside	329	131.1 (100%)	+	−	+	+	[22]
4	2.88	Coumaric acid	327/165	163.7 (100%)	+	−	−	−	[23,24]
5	2.95	Hydroxy benzoic acid isomer-1	137	121.0, 92.9 (100%)	−	+	−	−	[25,26]
6	3.59	gallic acid derivative	293	171.0 (100%)	−	+	−	−	[21]
7	4.91	Hydroxy benzoic acid isomer-2	137	94 [M+H−44], 77 [M+H−44-OH]	−	2.41	4.01	6.13	[25,26]
8	5.21	Caffeic acid	179	135.2 (100%) [M-H-COOH]	+	−	−	+	[24,25]
9	5.41	Gallic acid derivative	259	169.0	−	+	−	−	[21]
10	5.82	Vanillic acid derivative	279	167.4 (100%)	+	−	−	−	[22]
11	6.15	Chlorogenic acid isomer-1	353/355	191.3 (100%),137.3	+	−	−	+	[25,26]
12	6.43	Cinnamic acid derivative	329	146.6 (100%) [M−182]^−^	+	−	−	−	[22]
13	6.80	Caffeic acid derivative	371	178.9 (100%)	+	−	−	−	[24]
14	7.59	Quinic acid derivative	271	191.0 100%)	−	+	−	−	[22]
15	7.69	Ferulic acid derivative	273	192.7 (100%), 148.7 99	+	−	−	−	[24]
16	7.88	Hydroxyl gallic acid	185		1.99	−	−	−	[27]
17	8.50	Chlorogenic acid isomer 2	355		−	9.29	−	−	[25,26]
18	8.22	Caffeic acid derivative (malonyl rhamnoside)	367	135.0 (100%) [M-H−232]^−^	+	−	−	+	[24]
19	9.56	Ellagic acid	303	257.0, 229, 201.2, 164.9, 153.1, (100%)137.0, 123.0, 108.4	+	−	−	−	[28]
20	9.81	Quinic acid	193	119.0, 105.1, 91.0, 79	+	−	−	−	[28,29]
21	9.97	Sinapic acid isomer 1	225		6.35	−	−	−	[30]
22	10.13	Sinapicacid isomer-2	225		2.21	−	−	−	[30]
23	10.93	Methyl gallate isomer 1	185	171, 125	−	−	1.81	−	[20]
24	10.91	Methyl gallate isomer 2	185	171, 125	−	−	−	0.67	[20]
25	12.86	Coumaric acid derivative	279	162.6, 121.6 (100%)	−	+	−	−	[21]
26	13.95	Coumaric acid	163		−	−	2.95	−	[21]
27	15.29	Hydroxy ferulic acid	209		11.4	−	−	−	[31]
28	15.92	Benzoic acid derivative	307	120.8 (100%)	+	−	−	+	[21]
29	16.21	Chlorogenic acid isomer-3	353		−	−	9.69	−	[25,26]
30	16.55	Benzoic acid methyl ester	137		4.14	−	−	−	[21]
31	21.44	Quinic acid derivative	371	191.0 (100%)	−	+	−	−	[21]
32	22.31	Coumaric acid glucuronide	339	163.0	−	0.18	−	−	[21]

**Table 2:** Tentatively identified flavonoid compounds from the in vivo and in vitro tissues of *D. capillifolius* Gilli by using UPLC-ESI-MS/MS analysis in positive (+) and negative (−) ionization modes. (+) present, (−) absent.

No.	R_t_ (min)	Name	Parent Ion (*m/z)*	MS^2^ Fragments *(m/z)*	I	II	III	IV	Reference
**Flavonoid aglycones**									
33	13.56	Diosmetin	299/301	-ve/284.9 (100%) [M-CH_3_], 255.6 [M−15-CO]; or +ve/286.1, 258.0 (100%), 168.4, 146.9, 135, 130.0	+	−	−	−	[32]
34	11.77	Dimethoxyflavone	281	149.3 (100%) [ring A], 132.0 [ring B] 149(A)/132(B)	+	−	−	−	[33]
35	12.07	Luteolin	285	175.1, 132.8 (100%)	+	+	−	+	[34,35]
36	13.21	Apigenin	269	148.9, 119.2, 117.2 (100%)	+	−	−	−	[34]
37	16.20	Galangin	269/271	119.9 (100%), 152.9 (100%), 118.9	+	+	−	−	[25,36]
38	16.20	5,4′-dihydroxy-3,7-dimethoxyflavone	313	283.0 [M-H−30], 254.8 (100%) [M-H−30−28]	+	+	−	−	[33]
39	16.42	Methyl apigenin (Acacetin)	283	268.0 (100%) [M-H-CH_3_]	+	+	−	−	[33,37]
40	16.51	5-hydroxy-3′,4′,7-trimethoxy-flavanone	329	314, 299	−	2.51	−	3.11	[37]
41	17.32	Isorhamnetin	317		1.25	−	−	−	[20]
42	22.92	Dihydroxyflavone(chrysin)	255	135.0, 119.0(100%)	−	+	−	−	[33]
**Flavonoid-*O*-glycosides**									
43	1.76	Diosmetin-7-*O*-glucuronopyranosyl-*O*-rhamnoside	621	445.0 (100%) [M-H−176]	+	+	−	−	[38]
44	6.75	Myricetin-3-*O*-glucoside	479	317.0, 183.7, 161.1, 159.0	+	+	−	−	[39]
45	6.80	Myricetin-3-*O*- acetyl glucoside	491	317.1 (100%)	−	+	+		[40]
46	8.15	Quercetin-3-*O*-acetyl glucoside pentoside	639	303.0 (100%)	+	−	−	−	[41]
47	8.50	Quercetin diglucoside	625	301	1.08	−	−	−	[20]
48	8.75	Quercetin glucoside	463	301	1.83	−	−	−	[20]
49	9.14	Luteolin-7-*O*-rutinoside	593	285.2, 284.2 (100%)	+	−	−	−	[26,42]
50	9.25	Quercetin-*O*-rhamnoside	447	301	0.89	−	−	−	[20]
51	9.43	Apigenin-7-*O*-caffoeylhexoside	593	269.4 (100%)	+	−	−	−	[43]
52	9.43	Diosmetin-*O*-coumaroyhexoside	607	299.0 (100%), 163, 131.1	+	−	−	+	[44]
53	9.58	Quercetin-3-*O*-galactoside	463	300.6 (100%), 178.9	3.03	−	−	−	[24]
54	10.04	Apigenin-7-*O*-glucoside	431/433	268.0, 269.0 (100%), 108.0	+	+	−	+	[45]
55	10.14	Luteolin-7-*O*-glucoside	447	285.0 (100%)	+	+	−	−	[26,42]
56	10.32	Luteolin-7-*O*-glucuronoside	461	284.5, 283.3 (100%)	+	+	−	+	[26,32]
57	10.26	Apigenin-7-*O*-glucoside	431	270	0.77	−	−	−	[45]
58	10.40	Quercetin-O- rhamnoside	447	299.0	2.02		−	−	[20]
59	12.85	Diosmetin-*O*-rutinoside	609		2.22	−	−	−	[46]
60	13.27	Diosmetin-7-*O*-hexoside	461	299.3 (100%), 284.5	+	−	−	+	[47]
61	13.38	Luteolin derivative	567	285 (100%)	+	−	−	−	[34,35]
62	15.36	Luteolin acetyl glucoside	489	285	−	1.12	−	−	[34,35]
63	19.44	Diosmetin-7-*O*-rutinoside	609	300.9, 206.3 (100%), 157.4	−	−	−	−	[46]
64	21.91	Myricetin-3-*O*-rhamnoside	463	316.6 (100%)	+	+	+	+	[20]
**Flavonoid-*C*-glycosides**									
65	13.27	Diosmetin-8-*C*-rhamnoside	445	341 [M−104]^−^	+	−	−	+	[35,46]
66	23.27	Apigenin-8-*C*-hexoside	433	313.1 [M+H−120] 150.6, 130.7 (100%)	−	+	+	+	[48]
67	23.39	Diosmetin-8-*C*-glucoside	461	341.0 (M-H−120) (100%)	+	+	−	+	[46]
68	24.49	Diosmetin-8-*C*-glucoside-*O*-rhamnoside	609	489.2 [M+H−120], 462.5 [M+H−146], 341.9 [aglycone+H+41]	−	+	+	−	[48]

**Table 3:** Tentatively identified anthocyanins, tannins, and acetylenic and nitrogenous compounds from the in vivo and in vitro tissues of *D. capillifolius* Gilli by using UPLC-ESI-MS/MS analysis in positive (+) and negative (−) ionization modes. (+) present, (−) absent.

No.	R_t_ (min)	Name	Parent Ion (*m/z)*	MS^2^ Fragments *(m/z)*	I	II	III	IV	Reference
**Anthocyanins**									
69	6.36	Pelargonidin-3-*O*-glucuronosyl-*O*-glucoside	610	271.3	+	−	−	−	[49,50]
70	10.51	Cyanidin-3-*O*-glucoside	449	287.0	0.80	+	+	+	[51,52]
71	10.60	Cyanidin-3-*O*-glucoside	449	287.0	5.78	−	−	−	[51,52]
72	12.90	Cyanidin derivative	620	287(100%)	+	−	+	+	[49]
73	13.21	Cyanidin-*O*-glucuronosyl-*O*-glucoside Or feruloyl-*O*-glucoside	625	287.0 (100%)	+	−	−	+	[49,52]
74	16.95	Cyanidin derivative	721	287.4 (100%)	+	+	+	+	[49,52,53]
75	28.31	Malvidin-3-*O*-glucoside-malonyl-glucoside	741	331.4 [M−410]	+	+	−	+	[54]
**Tannins**									
76	7.17	Gallocatechin	305	261, 119, 97.0 (100%)	2.55	−	−	−	[55]
77	7.59	Epigallocatechin	305/307	261, 119, 97.0 (100%)	5.31	−	−	−	[55]
78	7.68	Epigallocotechin derivatives	721	304.7 (100%)	+	−	−	−	[55]
79	11.25	Catechin-3-*O*-hexoside-pentoside	585	294 [M−291]	−	−	+	−	[55]
80	14.54	Catechin-*O*-acetyl glucoside pentoside	625	288.5 (100%)	+	**−**	−	−	[50]
81	16.07	Catechin	291	174.9, 147.3, 137.3, 121, 106.9	−	−	−	−	[50]
**Acetylenic compounds**									
82	20.85	9-[Heptadeca-1-en-4,6,9-triyne-3,8-diol]	281	57.3, 56.7, 43.2, 54.9, 66.7, 79.0, 81.1, 95.0, 109.2, 83.1, 122.8, 71.0(100%)	+	+	+	−	[12,56,57]
83	23.45	11-[8-hydroxytetradeca-1-en-4,6,9-triyn-3-yl acetate]	281	97.0, 56.9, 80.9, 69.2, 95.4 106.6, 107.2 146.8 55.0 (100%)	+	+	+	−	[12,56,57]
84	28.27	Falcaridiol-8-*O*-methyl ether	297	167.3, 149.4, 134.9, 121.1, 106.8, 105, 97.0, 83.1 ,81.0, 69.1, 55.0 (100%), 42.8	+	+	+	−	[12,56,57]
**Nitrogenous compounds**									
85	1.35	3-Methyl-Indole	132	76.0	−	+11	18.55	17.6	[58]
86	1.77	4-(aminoethyl) benzoic acid	/166 or 120	119.9, 103.0 (100%), 93.0, 91.0, 76.9	−	+12	22.67	+	[59]
87	2.23	4-(aminoethyl) benzoic acid isomer	164/166	119.9, 103.0 (100%), 93.0, 91.0, 76.9	−	+20.9	1.82	21.57	[59]

**I**: methanol extract of cultivated fruit; **II:** methanol extract of callus grown on medium A; **III:** methanol extract of callus grown on medium B; IV: methanol extract of callus grown on medium C. (+) present, (−) absent.

Compounds **13** and **18** were readily detected at *m/z* of 371 and 367 [M-H]^−^, respectively. Based on the MS^2^ data in Table 1, they were tentatively identified as caffeic acid derivatives, as they produced the MS^2^ base peak fragment ions at *m/z* of 178.9 [M-H−192]^−^ and 135.0 [M-H−232]^−^ (possibly malonyl rhamnoside), respectively. Several caffeic acid derivatives have been previously reported in the genus *Daucus* [41].

Compounds **14** had ESI-MS with a deprotonated molecular ion at *m/z* of 271, which fragmented in MS^2^ to produce a base peak fragment ion at *m/z* of 191.0 which was identified as quinic acid derivative [22].

Compound **15** (R_t_7.69 min), a ferulic acid derivative, was determined with MS^1^ [M-H]^−^ at *m/z* of 273 and an MS^2^ base fragment ion at *m/z* of 192.7. Several ferulic acid derivatives were detected in the genus *Daucus* [24].

Compound **16** with molecular ion peak at *m/z* of 185 [M-H]^−^ was tentatively identified as hydroxy gallic acid [27].

Compound **19** showed a molecular ion peak at *m/z* of 303 [M+H]^+^ and was tentatively identified as ellagic acid. The MS^2^ showed typical fragmentation of ellagic acid at *m/z* of 257.0, 229.0, 201.2 and 164.9 and a base peak fragment at 153.1 [20,28]. Ellagic acid has gained a lot of interest due to its anti-inflammatory, antitumor antibacterial and liver protection effects [60].

Compound **20** had a protonated molecular ion fragment at *m/z* of 193 and gave an MS^2^ fragment at *m/z* of 119.0. This compound was identified as quinic acid [28]. Notably, it was previously reported in the genus *Daucus* [24].

Compounds **21** and **22** were identified as sinapic acid isomers 1 and 2, based on the presence of the molecular ion peak at *m/z* of 225 [30].

Compounds **23** and **24** were identified as isomers 1 and 2 of methyl gallic acid based on the molecular ion peak at *m/z* of 185 as reported by [20]. Meanwhile, compound **26** (R_t_ of 13.95) was identified as coumaric acid, supported by the presence of a molecular ion peak at *m/z* of 163 [M-H]^−^ as reported by [21].

Compound **27** was tentatively identified as hydroxy ferulic acid according to [31] and was detected from the ion fragment at *m/z* of 209 [M-H]^−^. Further, compound **30** was identified as benzoic acid methyl ester after the detection of the ion fragment at *m/z* of 137 [M-H]^−^ according to [21].

Finally, compound **32** was identified as coumaric acid glucuronide based on the presence of a molecular ion fragment at *m/z* of 339 [M-H]^−^ and the fragment ion at 163 comprising coumaric acid after the loss of the glucuronic acid moiety [M-H−176]^−^ [21].

#### 2.1.2. Identification of Flavonoids

##### Flavonoids Aglycones

Ten flavonoid aglycones were identified in methanol extracts of in vivo and in vitro tissues, as well as in the fruit extract of the cultivated plant *Daucus capillifolius*, as described in the following.

Compound **33** (R_t_ of 13.56 min) was identified as diosmetin from the ESI-MS spectrum, which showed deprotonated and protonated molecular ions at *m/z* of 299 and 301, respectively. The ESI-MS/MS fragmentation pattern showed fragment ions at *m/z* of 284.9 (100%) [M^+^-H-CH_3_] and 255.6 [M^+^-CH_3_-CO], in addition to the fragment ions mentioned in Table 3, which are characteristic of fragmentation for diosmetin [32]. Flavones such as diosmetin and apigenin were reported in *Petroselinum crispum*, Apiaceae. It was used in menstrual disorders treatment due its phytoestrogen content [14]

Compound **34** produced a mass spectrum [M^+^-H] at *m/z* of 281. It was identified as 5,4-dimethoxyflavone through MS^2^ fragment ions at *m/z* of 149.3 (100%) and 132.0, which are characteristic for ring A and ring B, respectively, each with one methoxy group [33].

Compound **35** (R_t_ of 12.07 min) was identified as luteolin based on the [M^+^-H] at *m/z* of 285 and the MS^2^ fragmentation pattern presented in Table 2, as reported by [34]. It is worth noting that luteolin has been previously isolated from *Daucus* species [61].

**Figure 4:**
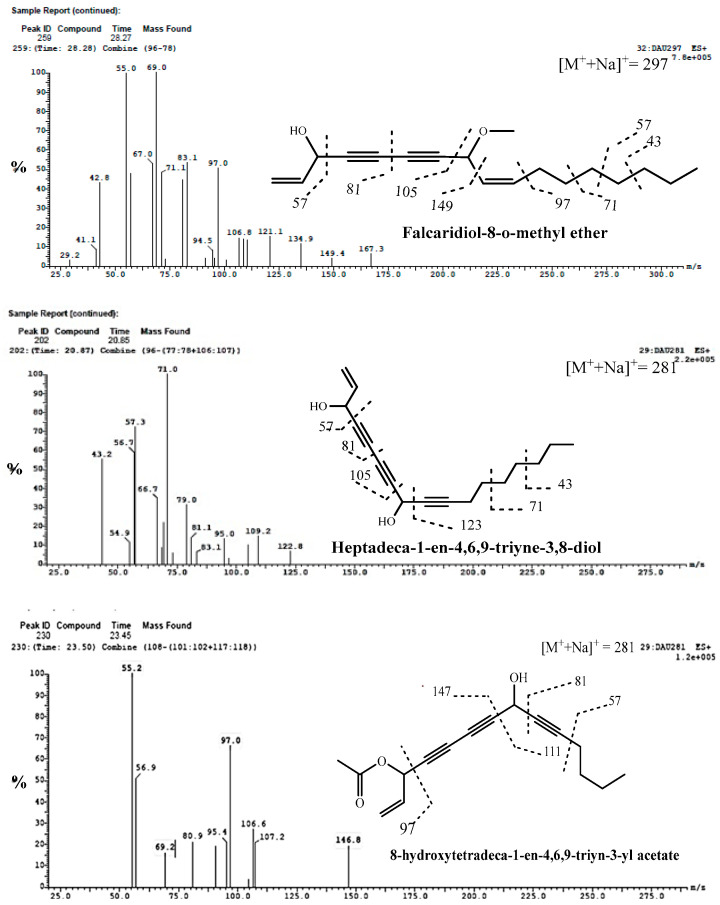
Fragmentation pattern mass spectra of some identified acetylenic compounds from methanolic extracts of cultivated fruits and calli grown on different media of *Daucus capillifolius* Gilliin in positive (+) and negative (−) ionization modes.

Compounds **36** and **37** were identified as apigenin and galangin, respectively, from the fragmentation pattern in both positive and negative modes (Figure 4) of ESI-MS/MS, as shown in Table 2 [25,34,36].

Compound **38** was identified as 5,4′-dihydroxy-3,7-dimethoxyflavone according to the comparison with previous studies [33]. It had a molecular ion at *m/z* of 313 [M^+^-H]^−^, which produced MS^2^ fragment ions at *m/z* of 283.0 [M^+^−30 (OCH_3_)]^−^ and 254.8 [M^+^-H−30−28 (OCH_3_+CO)] (100%) [33].

**Figure 5:**
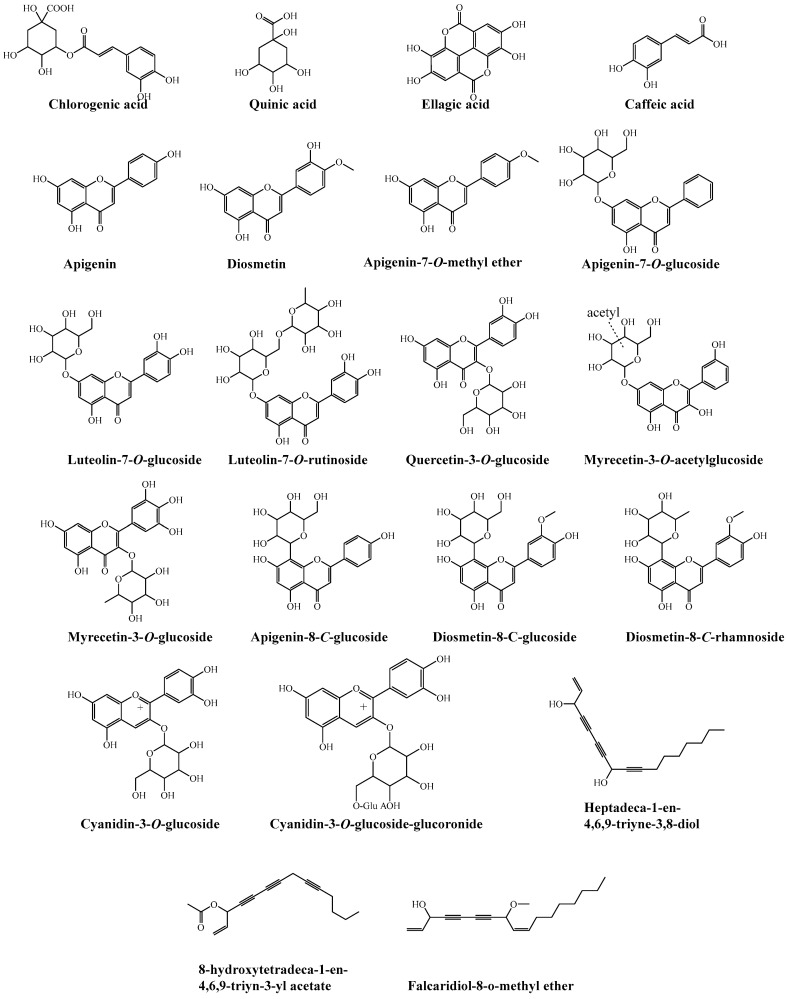
Chemical structures of the tentatively identified compounds from methanolic extracts of cultivated fruits and calli grown on different media of *D. capillifolius* Gilli.

Regarding compound **39**, its ESI-MS spectrum (Figure 5) showed a molecular ion compound at *m/z* of 283 [M−H]^−^ with an MS^2^ fragment ion at *m/z* of 268.0 [M-H−15]^−^. Therefore, the compound was identified as apigenin-4′-methyl ether (acacetin) [33,37].

Compound **40** showed an [M^+^-H]^−^ ion at *m/z* of 329, with the production of daughter ions at *m/z* of 314 and *m/z* of 299, indicating the loss of two methyl groups from the parent 329 ion. Therefore, it was tentatively identified as 5-hydroxy-3′,4′,7-trimethoxy-flavanone [33,37].

Compound **41** was tentatively identified as isorhamnetin, as the MS/MS spectrum of this compound showed the characteristic product ion at *m/z* of 317 [M^+^−H] [33,37].

Compound **42** (R_t_ of 22.92 min) was identified as a dihydroxy flavone from its ESI-MS spectrum with [M^+^+H] at *m/z* of 255. MS^2^ fragmentation showed the presence of one hydroxyl group in both of rings **A** and **B**, where it showed a base compound fragment ion at *m/z* of 119.0 [M^+^+H−136] and a fragment at *m/z* of 135.0 [33].

##### Identification of Flavonoid Glycosides

Identification of *O*-glycosides

Twenty-two flavone or flavonol-*O*-glycosides were identified in methanol extracts of in vivo and in vitro tissues of *D. capillifolius*, as described below.

Compound **43** was identified asdiosmetin-7-*O*-glucuronopyranosyl-*O*-rhamnoside according to the ESI-MS data reported in Table 2, which show a pseudo-molecular ion at *m/z* of 621 [M^+^-H]^−^ and an MS^2^ fragment ion at *m/z* of 445.0 [diosmetin-7-*O*-rhamnoside-H], which shows the loss of 176 amu [38].

Compound **44** exhibited a molecular ion compound at *m/z* of 479 [M-H]^−^. The fragment ions in MS^2^ at *m/z* of 317.0 [M-H−162]^−^ showed the loss of a hexose moiety. Additionally, other fragment ions at *m/z* of 161.1 and 159.0 were observed. Based on these findings, this compound was identified as myricetin-3-*O*-glucoside, as depicted in Figure 5 [39].

Compound **45** presented a molecular ion at *m*/*z* of 491 [M-H]^−^. The MS data show a base fragment signal at *m*/*z* of 317.1 [M-H−204]^−^, indicating the loss of acetyl hexoside. From these results, compound **45** was tentatively identified as myricetin-*3-O*-acetylglucoside [40].

Compound **46** was identified as quercetin acetyl glucoside pentoside based on the [M+H]^+^ ion at *m/z* of 639 and at *m/z* of 303.0 (100%) [quercetin+H]^+^, which indicates a loss of acetylglucose and pentose moieties [204+132] [41].

Compounds **47**, **48** and **50** exhibited pseudo-molecular ions at *m/z* of 625, 463 and 447, respectively. Through analysis of the MS/MS spectrum, these compounds displayed a characteristic product ion at *m/z* of 301, corresponding to quercetin. This product ion resulted from the loss of diglucoside [M^+^-H−324] for **47**, a glucosyl [M^+^-H−162] for **48** and a rhamnosyl [M^+^-H−146] moiety for **50**. As a result, these compounds were identified as quercetin diglucoside, quercetin-*O*-glucoside (Figure 5) and quercetin-*O*-rhamnoside [41].

Compounds **49** and **56** (R_t_ of 9.14 and 10.32 min) exhibited molecular ion peaks at *m/z* of 593 and 461 [M^+^-H]^−^, respectively, and MS^2^ fragment ions at *m/z* of 285.2 and 284.5 [luteolin-H]^−^ after the neutral loss of rutinose [M^+^-H−308]^−^ and glucuronide [M^+^-H−176]^−^ moieties, respectively. The loss of 176 amu is characteristic of a glucuronic acid moiety [24]. Therefore, these compounds were identified to be luteolin-7-*O*-rutinoside (**49**) and luteolin-7*-O*-glucoronoide (**56**), respectively [42]. Luteolin-7*-O*-glucoronoide was previously isolated from the genus *Daucus* [26].

Compounds **51**, **52** and **53** with R_t_ of 9.43 and 9.58 min gave deprotonated molecules for three compounds at *m/z* of 593, 609 and 463 [M^+^-H]^−^, respectively. The MS^2^ base fragment ions at *m/z* of 269.4, 299.0, and 300.6 for [Apigenin−H]^−^, [diosmetin−H]^−^ and [quercetin−H]^−^, respectively, showed the neutral loss of caffoeylhexoside [M^+^−H−324]^−^, coumaroyl hexoside [M^+^−H−308]^−^ and galactoside moieties [M−H−162]^−^, respectively. From the previous results and as shown in Table 2, these compounds were identified to be apigenin-7-*O*-caffoeyl hexoside (**51**) [43], diosmetin-7-*O*-coumaroyhexoside (**52**) [44] and quercetin-3-*O*-galactoside (**53**). Compound **53** was previously reported in *Daucus* species [24].

The ESI-mass spectra of compounds **54** and **55** (Figure 3) exhibited deprotonated molecules at *m/z* of 431 and 447 [M^+^−H]^−^, respectively, and MS^2^ base compound fragment ions at *m/z* of 269.0 [apigenin-H]^−^ and 285.0 [luteolin-H]^−^ due to the neutral loss of a glucose moiety [M-H−162]. These compounds were identified to be apigenin 7-*O*-glucoside and luteolin-7-*O*-glycoside, respectively [42]. Both compounds were previously isolated from the genus *Daucus* [26,61].

Compound **57** was identified as an isomer of compound **54** and identified as an apigenin 7-*O*-glucoside isomer. Similarly, compound **58** (R_t_ of 10.40 min) showed molecular ions at *m/z* of 447 and another fragment ion at *m/z* of 299, which corresponds to [M^+^-H] after the loss of the rhamnosyl moiety. It was identified as an isomer of compound **50** and identified as a quercetin-*O*-rhamnoside isomer.

Compounds **59** and **63** were proposed as isomers of diosmetin-*O*-rutinoside. They exhibited molecular ions at *m/z* of 609 [M+H]^+^. The product ion in the MS/MS spectrum was at *m/z* of 300.9 [M^+^+H−308], showing loss of the rutinose moiety [46].

Compound **60** (R_t_ of 13.27 min) was tentatively identified as diosmetin-*7-O*-glucoside which was previously isolated from the genus *Daucus* [61]. This identification was based on the ESI-MS spectrum, which presented a molecular ion peak at *m/z* of 461 [M^+^-H]^−^, and the MS^2^ data that show a fragment ion at *m*/*z* of 299.3 [diosmetin-H], indicating a loss of the glucose moiety [47].

Compounds **61** and **62** had molecular ion fragments at 567 [M^+^-H] and 489 [M^+^-H], respectively. Upon analyzing the fragmentation pattern, MS^2^ fragment ions at *m/z* of 285.0 (100%) [M^+^-H−282]^−^ were observed for **61**, indicating a loss of 282 atomic mass units. For **62**, the fragmentation pattern displayed 285 [M^+^-H−204]^−^, indicating the loss of an acetyl hexoside. Consequently, compound **61** was identified as a luteolin derivative, while compound **62** was identified as luteolin–*7*-*O*-acetyl hexoside [41]

Compound **64** exhibited a deprotonated molecular ion compound at *m/z* of 463 and was tentatively identified as myricetin-3-*O*-rhamnoside, as it gave the MS^2^ base compound fragment ion at *m/z* of 316.6, corresponding to the neutral loss of the rhamnose moiety (146 amu) [20].

Identification of C-glycosides

Four flavone *C*-glycosides (Table 2) were identified in methanol extracts of in vivo and in vitro tissues of *D. capillifolius* Gilli, as described below.

Compound **65** (R_t_ of 13.27 min) had the molecular ion peak in the ESI-MS spectrum at *m/z* of 445 [M^+^-H]^−^ and an MS^2^ fragment ion at *m/z* of 341.0 [M−104]^−^, which showed the loss of 104 amu characteristic for the 8-C-rhamnoside of flavone [46,48]. Therefore, compound **65** was concluded to be diosmetin-8-*C*-rhamnoside (Figure 3). It is the first report of compound **65** in the genus *Daucus*. Diosmetin-di-*C*-rhamnoside was previously isolated from *Daucus carota* [35].

Compound **66** (R_t_ of 23.27 min) displayed a molecular ion compound at *m/z* of 433 [M+H]^+^ and an MS^2^ fragment ion at *m/z* of 313.1 [M+H−120] corresponding to the ^0.3^X ion. Another fragment ion signal at *m/z* of 150.6 and 130.7 (100%) was observed, which, along with the previous fragment, is characteristic for apigenin-8-*C*-glucoside [48]. 

Compound **67** (R_t_ of 23.39 min) presented a molecular ion at *m*/*z* of 461 [M^+^-H]^−^. The MS^2^ data show a fragment signal at *m/z* of 341.0, indicating the loss of 120 amu (^0.3^X ion). This fragment for [aglycone + 41] is a characteristic feature of mono *C*-glucoside flavonoid. Moreover, the absence of a fragment at [M^+^-H−18] indicates the presence of 8-*C*-glucoside instead of 6-*C*-glucoside [46]. Compound **67** was tentatively concluded to be diosmetin-8-*C*-glucoside (Figure 3) [46].

Compound **68** was suggested to be diosmetin-8-*C*-glucoside-*O*-rhamnoside. This was confirmed by the [M+H]^+^ ion at *m/z* of 609 and by the MS^2^ compound at *m/z* of 489.2, which indicates a loss of 120 amu (^0.3^X ion), a characteristic feature of a *C*-glucoside flavonoid. The fragment at *m/z* of 462.5 [M^+^+H−146] and at *m/z* of 341.9 [aglycone+H+4] for mono *C*-glycoside flavonoid and the absence of a fragment at [M^+^-H−18] indicated the presence of 8-*C*-glycoside rather than 6-*C* glucoside [48].

#### 2.1.3. Identification of Anthocyanins

Six anthocyanin compounds were identified in methanol extract of in vivo and in vitro tissues of *D. capillifolius* Gilli. They were identified as glycosides or acylated glycosides of cyanidin, pelargonidin and malvidin, as shown in Table 3 and described below.

Compound **69** exhibited a molecular ion peak [M+H]^+^ at *m/z* of 610, which on MS^2^ produced a fragment ion at *m/z* of 271.3 corresponding to pelargonidin aglycone [M^+^+H −338] with the loss of glucose and glucuronide moieties [50]. From the previous results, compound **69** was identified as pelargonidin-3-*O*-glucuronosyl-*O*-glucoside. The position of the glucouronide moiety could not be identified.

Compounds **70** and **71** had a [M]^+^ at *m/z* of 449 (Figure 3), which on MS^2^ produced an ion at *m/z* of 287 (cyanidin, [M^+^−162]), with the loss of a glucose moiety. From the previous results, compounds **70** and **71** were identified as isomers of cyanidin-3-glucoside [51].

Compound **72** had [M^+^+H] at *m/z* of 620 which on MS^2^ produced an ion at *m/z* of 287.0 (100%) (cyanidin, [M^+^+H−332]). From the previous results, compound **72** was suggested to be a cyanidin derivative [49].

Compound **73** was suggested to be cyanidin-*O*-glucuronosyl-*O*-glucoside (Figure 3) or cyanidin-*O*-feruloylglucoside. This was confirmed by the [M^+^] ion at *m/z* of 625 and by the MS^2^ compound fragment at *m/z* of 287.0 (100%), indicating the loss of the glucuronosyl-*O*-glucoside moiety or feruloyl glucoside moiety [49,52].

Compound **74** was also suggested to be a cyanidin derivative. This was confirmed by the [M^+^] ion at *m/z* of 721 and by the MS^2^ compound ion at *m/z* of 287 (100%) [49,52].

Compound **75** (R_t_ of 28.31 min) was suggested to be malvidin-3-*O*-glucoside malonyl glucoside, which was confirmed by the [M^+^] ion at *m/z* of 741 and by the MS^2^ compound at *m/z* of 331.4 [M^+^−410] indicated the loss of glucoside malonylglucoside [54]. Anthocyanins of different cultivars of black carrot are relatively stable under low-acid conditions and could be used as natural food-coloring agents [24]

#### 2.1.4. Identification of Tannins

Six flavanes compounds were identified in methanol extracts of in vivo and in vitro tissues of *Daucus capillifolius* as shown below.

Compounds **76**, **77** and **78** exhibited characteristic features in their mass spectra. Compound **76** (gallocatechin) displayed [M^+^-H]^−^ at *m/z* of 305 and MS^2^ fragments at *m/z* of 261.0, 119.0 and 97.0 (100%). Similarly, compound **77** (epigallocatechin) exhibited [M^+^-H]^−^ at *m/z* of 305 and [M+H]^+^ at *m/z* of 307 and shared the same MS^2^ fragment ions as compound **76**. On the other hand, compound **78**, an epigallocatechin derivative, showed [M^+^-H]^−^ at *m/z* of 721 and a specific MS^2^ fragment ion at *m/z* of 304.7 [55].

Compound **79** (R_t_ of 11.25 min) was suggested to be catechin-3-*O*-hexosidepentoside, which was confirmed by the [M+H]^+^ ion at *m/z* of 585 and by the MS^2^ compound at *m/z* of 294 [M^+^−291]^−^ which indicates a loss of 291 amu [catechin+H] (Table 3) [55]. The fragment at *m/z* of 294 [162+132] is a characteristic feature of hexose and pentose moieties.

Compounds **80** and **81** displayed distinct molecular ion signals. Compound **80** exhibited an [M-H]^−^ signal at *m/z* of 625, while compound **81** showed an [M+H]^+^ signal at *m/z* of 291. The fragmentation pattern for compound **80** revealed MS^2^ fragments at *m/z* of 288.5 (100%), indicating a loss of 336 atomic mass units. On the other hand, compound **81** displayed MS^2^ fragments at *m/z* of 174.9, 147.3, 137.3, 121.0 and 106.9, which are typical fragmentation patterns associated with catechin. As a result, compound **80** was identified as catechin-*O*-acetyl glucoside pentoside, while compound **81** was identified as catechin. These compounds are not common in daucus species but have been isolated and identified in green tea [50].

#### 2.1.5. Identification of Acetylenic Compounds

Compounds **82** and **83** were tentatively identified as 9-[Heptadeca-1-en-4,6,9-triyne-3,8-diol] and 11-[8-hydroxytetradeca-1-en-4,6,9-triyn-3-yl acetate], respectively. From MS^1^ data, the compounds showed the same molecular ion peaks at *m/z* of 281 [M+Na]^+^ and characteristic fragmentation patterns as shown in Table 3 and Figure 4 at *m/z* of 81, 91, 97, 105, 123, 147, 149, 111, 71, 69, 71 and 57 [12,56,57].

Compound **84** showed predominantly sodiated ions and no [M^+^+H] ions [57]. The ESI-MS spectrum (Figure 4) showed a molecular ion compound at *m/z* of 297 [M+Na]^+^ and MS^2^ fragments ions at *m/z* of 81, 105, 149 and 167, which are typical fragmentation patterns of falcarindiol-*O*-methyl ether. Consequently, it was identified as falcarindiol-8-*O*-methyl ether. Falcarinol, falcarindiol and falcarinone were previously reported in the genus Daucus [12,56,57].

In the extract obtained from the calli grown in media A and C, falcarindiol-8-*O*-methyl ether (**84**) was present at a higher concentration. Conversely, it was found at a lower concentration in medium B and cultivated fruits. On the other hand, compound **82** showed a higher concentration in the extract obtained from cultivated fruits compared with the calli grown on the three types of media, where it was present in very minimal amounts. Compound **83** was found in minor quantities in all the extracts.

#### 2.1.6. Identification of Nitrogenous Compounds

Compound **85** (R_t_ of 1.35 min) exhibited protonated molecular ions [M^+^+H]^+^ with an *m/z* value of 132 with a fragment ion at *m/z* of 76, which are characteristic of 3-methyl indole [58].

Compounds **86** and **87** (R_t_ of 1.77 and 2.23 min) were tentatively suggested to be 4-(aminoethyl) benzoic acid isomers based on the presence of a protonated molecular ion fragment at *m/z* of 166 [M+H]^+^ along with another fragment at *m/z* of 119.9 [M^+^+H-COOH] and a base peak fragment at *m/z* of 103 [119.9-NH_2_]^+^, in addition to other characteristic fragments at *m/z* of 93.0, 91.0 and 76.9 [59]. Notably, this is the first report about the presence of nitrogenous compounds in this genus.

## 3. Materials and Methods

### 3.1. Plant Materials

The fruits of the cultivated *Daucus capillifolius* Gilli plant were collected in the fruiting stage in 2016 from the Farm of the Pharmacognosy Department, Faculty of Pharmacy, Zagazig University, Zagazig, Egypt. The plant was kindly identified by the late Prof Dr. Hussein Abdel Basset, Professor of Taxonomy, Faculty of Science, Zagazig University. A voucher specimen (D.C 2016/12) was deposited at the herbarium in the Department of Pharmacognosy, Faculty of Pharmacy, Zagazig University, Egypt. *D. capillifolius* fruits were air-dried and ground into coarse particles for use. Additionally, 50 g of 100-day-old calli grown on three different media, medium A [M&S + NAA (1 mg/L) + BAP (0.1 mg/L)], medium B [M&S + TDZ (0.5 mg/L) + 2,4 D (1 mg/L) + BAP (0.1 mg/L)] and medium C [M&S + 2, 4D (2 mg/L) + K (1 mg/L)], was prepared from the seedling explants of *D. capillifolius* fruits for the analysis.

#### 3.1.1. Induction of Calli from In Vitro Germinated Seedlings

Callus was initiated from leaf explants of *D. capillifolius* seedlings as described by [19]. Excellent growth of calli with friable greenish white, friable bright yellow and compact yellowish white was obtained from calli grown on media A [M&S + NAA (1 mg/L) + BAP (0.1 mg/L)], B [M&S + TDZ (0.5 mg/L) + 2,4D (1 mg/L) + BAP (0.1 mg/L)] and C [M&S + 2,4 D (2 mg/L) + Kinetin (1 mg/L)], respectively (Figure 1).

#### 3.1.2. Extract Preparation

Air-dried powdered fruits of *D. capillifolius* Gilli (200 g) were extracted by using methanol (HPLC analytical grade), filtered by using a membrane disk filter (0.2 µm) and then subjected to LC-ESI-MS analysis. A total of 50 g of 100-day-old non-organic calli grown separately on MS media with different hormonal compositions, including media A [M&S + NAA (1 mg/L) + BAP (0.1 mg/L)], B [M&S + TDZ (0.5 mg/L) + 2,4 D (1 mg/L) + BAP (0.1 mg/L)] and C [M&S + 2,4 D (2 mg/L) + Kinetin (1 mg/L)], was extracted with HPLC methanol (100 mL × 3). The extracts were collected and dried under vacuum by using a rotary evaporator at a temperature not exceeding 60 °C to give four extracts kept at 4 °C till analysis.

### 3.2. UPLC-ESI-MS/MS Analysis and Separation Method of D. capillifolius Extracts

UPLC-ESI-MS/MS (ultra-performance liquid chromatography–electrospray tandem mass spectrometry) in both ionization modes was carried out as described by [62] on an aXEVO-TQD triple-quadruple instrument (Waters Corporation, Milford, MA, USA) mass spectrometer (ACQUITY UPLC BEH C18 (1.7 μm, 2.1–50 mm) column; column flow rate of 0.2 mL/min). The solvent system consisted of (A) water and (B) methanol, both containing 0.1% formic acid (Ain Shams University, Cairo, Egypt). The gradient was programmed as follows: 0 min, 10% B; 5 min, 30% B; 15 min, 70% B; 22 min, 90% B; 25 min, 90% B; 26 min, 100% B; 29 min, 100% B; 32 min, 10% B. Finally, the initial conditions were held for 3 min as a re-equilibration step. The flow rate was 0.2 mL/min, and the sample at a concentration of 100 g/ml was prepared in HPLC-grade methanol, degassed, and filtered by using a 0.2 µm membrane disc filter before being subjected to LC-ESI-MS analysis. The injection volume was 10 µL. The parameters for analysis in negative ion mode were as follows: source temperature of 150 °C, cone voltage of 30 eV, capillary voltage of 3 kV, desolvation temperature of 440 °C, cone gas flow of 50 L/h and desolvation gas flow of 900 L/h. Mass spectra were detected in the ESI negative and/or positive ion modes between 50 *m/z* and 900 *m/z*. The peaks and spectra were processed by using MassLynx 4.1 software and tentatively identified by comparing their retention time (R_t_) and mass spectrum with the reported data. A fragmentation collision energy of 40 eV was used.

## 4. Conclusions

*Daucus capillifolius* Gilli, grown in Libya, is an endangered plant. Its micropropagation and callus culture were successfully established in our previous work with GC-MS analysis of its essential oil. In the current study, we investigated its phytoconstituents for the first time by using UPLC-ESI-MS/MS analysis. Our results revealed that *D. capillifolius* fruit extract is a rich source of phenolic compounds, including simple phenolic acids, anthocyanidins, tannins, flavonoids, flavonoids -*O*- and -*C*-glycoside, and acetylenic compounds. Moreover, the extracts from the in vitro calli grown on media A, B and C with different hormonal combinations showed the accumulation of less phenolic acids, acid derivatives tannins, compared with the cultivated fruit extract. All the tested extracts exhibited the formation of acetylenic compounds, but only the extracts of the in vitro calli. showed the accumulation of nitrogenous compounds. Notably, only luteolin was detected in the extract of the in vitro calli grown on medium C, while calli grown on medium B did not show any flavonoidal aglycons. In summary, this variation in the accumulation of secondary metabolites based on the investigated hormonal combination requires further future studies to achieve the required amounts of secondary metabolites compared with the wild and cultivated *D. capillifolius* plant.

## Data Availability

The data presented in this work are available in the article.
